# Trends in Disability Among Adults 55-64 in the United States and England From 2002 to 2016

**DOI:** 10.1093/geroni/igaa057.970

**Published:** 2020-12-16

**Authors:** HwaJung Choi, Robert Schoeni, Tsai-Chin Cho, Kenneth Langa

**Affiliations:** 1 University of Michigan, Ann Arbor, Michigan, United States; 2 University of Michigan, Ann Arbor, United States

## Abstract

The paper’s goal is to assess whether and, if so, the extent to which prevalence in disability of adults near retirement ages in the US increased over time compared to their peers in England and examine income group differences in the relative trends. This study uses 2002-2016 Health and Retirement Study (HRS) and English Longitudinal Study of Ageing (ELSA) focusing on adults aged 55-64. Annual percent changes over the period of 2002-2016 for limitations in instrumental activities of daily living (IADL) and activities of daily living (ADL) are estimated for each survey (HRS and ELSA) using multivariable logistic regressions to adjust for individual-level characteristics While disability prevalence of adults ages 55-64 in England improved over the years of 2002-2016 (annual % change= -2.01 for IADL; - 2.53 for ADL), disability prevalence of US adults has not improved and in fact even worsened in terms of IADL (annual % change= +1.35). There are substantial variations in the IADL/ADL trends by income groups. In the US, the adverse trends in disability were more pronounced among the lowest income groups (annual % change in IADL=1.76 for bottom 20% vs. -2.08 for top 20%; annual % change in ADL=1.08 for bottom 20% vs. -2.08 for top 20%). In England, the disability status improved over time for all but the lowest income group. We will examine further to identify specific factors contributing to divergent/convergent trends in disability between the US and England.

